# Quality of Electronic TB Register Data Compared with Paper-Based Records in the Kyrgyz Republic

**DOI:** 10.3390/tropicalmed8080416

**Published:** 2023-08-16

**Authors:** Daniil Shauer, Ofelya Petrosyan, Manik Gemilyan, Edward M. Kamau, Pruthu Thekkur, Olga Goncharova, Kalmambetova Gulmira, Bolot Kyrbashov, Kylychbek Istamov, Meder Kadyrov, Ewan Wilkinson

**Affiliations:** 1 National Center for Tuberculosis, Ministry of Health, Bishkek 720000, Kyrgyzstan; goncharova.ncph@gmail.com (O.G.); gulmirakalmambetova@gmail.com (K.G.); bolotkyrbashov@gmail.com (B.K.); mederkadyrow@gmail.com (M.K.); 2 TB Research and Prevention Center, Yerevan 0014, Armenia; ophelia.petrosian@gmail.com (O.P.); mgemilyan@yahoo.co.uk (M.G.); 3 Department of Gastroenterology, Yerevan State Medical University, Yerevan 0025, Armenia; 4 UNICEF/UNDP/World Bank/WHO Special Programme for Research and Training in Tropical Diseases (TDR) World Health Organization, 1211 Geneva, Switzerland; kamaued@who.int; 5 Centre for Operational Research, International Union Against Tuberculosis and Lung Disease, 2 Rue Jean Lantier, 75001 Paris, France; pruthu.tk@theunion.org; 6 School of Medicine, Osh State University, Osh City 723500, Kyrgyzstan; istamovk@gmail.com; 7 The Institute of Medicine, University of Chester, Chester CH14BJ, UK; ewan.wilkinson@gmail.com

**Keywords:** Kyrgyz Republic, drug-sensitive pulmonary TB, electronic register system, paper-based records, completeness, concordance, treatment initiation, treatment outcomes, SORT IT, operational research

## Abstract

This study evaluated the effectiveness of an electronic system for managing individuals with drug-sensitive pulmonary tuberculosis in the Kyrgyz Republic. This cohort study used programmatic data. The study included people registered on the paper-based system in 2019 and 302 people registered on both the electronic and the paper-based systems between June 2021 and May 2022. The data from the 302 individuals were used to assess the completeness of each form of record and the concordance of the electronic record with the paper-based system. This study showed that for most variables, the completeness and concordance were 85.3–93.0% and were lowest for nonmandatory fields such as medication side effects (26.8% vs. 13.6%). No significant difference was observed in the time taken from symptom onset to diagnosis and treatment initiation between the two systems. However, the electronic system had a significantly higher percentage of subjects who initiated treatment on the day of diagnosis (80.3% vs. 57.1%). The proportion with successful outcomes was similar in both groups, but the electronic system had a significantly lower proportion of individuals with outcomes that were not evaluated or recorded (4.8% vs. 14.3%, *p* < 0.001). This study highlights the potential advantages and gaps associated with implementing an electronic TB register system for improving records.

## 1. Introduction

In the Kyrgyz Republic, 2019 was declared the year of digitalization by the government. This initiative aimed to integrate innovative electronic technologies into various sectors, including healthcare. Significant progress in controlling tuberculosis (TB) globally has been made since 2005, but this was not maintained during the COVID-19 pandemic [1]; TB remains a major public health challenge in the Kyrgyz Republic, where around 5000 new cases are diagnosed annually [2]. Effective and systematic antituberculosis treatment is essential for those diagnosed with TB to ensure positive health outcomes. However, there is a need to develop and implement effective systems to track and monitor people with TB and their progress through the healthcare system.

Data on people with TB are used to monitor different aspects of the management of national TB control programs, including identifying delays in treatment initiation. This information helps those responsible for managing programs to improve patient care by addressing issues related to management such as the time between diagnosis and initiation of treatment. By tracking treatment outcomes and comparing them with those of other national programs, program administrators can take the necessary steps to address issues related to TB management. By understanding the duration of treatment episodes and identifying delays in treatment, healthcare professionals can better manage individual treatment outcomes and ensure that the program’s resources are used efficiently.

The World Health Organization (WHO) has been encouraging the adoption of digital technologies in the management of TB programs, including electronic registers, since 2015 [3]. Digital technologies are critical components for creating sustainable health systems and achieving universal health coverage. There are five main areas where electronic technology can contribute to TB control: (i) patient care and electronic directly observed therapy, (ii) surveillance and monitoring, including health information system management, (iii) measuring the burden of TB disease and death, (iv) monitoring drug resistance and program management issues such as stock control, and (v) electronic learning [4].

To support the digitalization initiative in the Kyrgyz Republic, a medical information system (MIS) was developed for tuberculosis services. This system includes an electronic health record (EHR), a laboratory information system (LIS), and an electronic TB register. The integration of these systems has eliminated the need for entering the same data into several systems, reducing errors and improving data quality. Additionally, this integration has contributed to the timely receipt of results, which could potentially minimize delays in treatment initiation. In penitentiary facilities in the Kyrgyz Republic, treatment delays (more than ten days delay compared with a mean of seven days) have been reported in around 47% of cases [5]. It is important to emphasize that in both scenarios—whether there is a delay between symptom onset and treatment initiation or between diagnosis and treatment initiation—the delay primarily arises from individuals with TB actively deciding to commence treatment. This delay is driven by human factors such as accepting the diagnosis and navigating the process of engaging with the healthcare facility. However, it’s equally important to note that treatment delays can also arise from broader health system challenges. These could involve delays in diagnosis beyond patients’ control due to factors such as inadequate resources for rapid diagnostics, and logistical issues within the healthcare infrastructure, like transportation barriers. While patient-related factors are crucial, treatment delays result from a mix of patient decisions and healthcare system obstacles.

Studies have shown that using electronic register systems in low-income countries can have a positive impact on patient care quality, management, and planning [6]. Using an electronic TB register can improve the accuracy and completeness of the data recorded for each person, potentially improving treatment outcomes. While it may be expected that changing from a paper-based system to an electronic register system would improve data quality, experience elsewhere has shown that implementing the change can be challenging. The determinants of success (or failure) include ethical, financial, technical, organizational, and training-related issues [7]. Some studies have shown that the quality of the data improves [8] and the time from diagnosis to treatment initiation is reduced [9]. However, others have found that where both paper-based and electronic systems are used in parallel, neither system contains high-quality data [10]. This further highlights the importance of avoiding dual recording of data to ensure reliable information.

The electronic TB register was introduced in the Kyrgyz Republic in 2020 but has not yet been evaluated. This study aimed to assess the completeness and reliability of the data in both the paper-based records and the electronic register and evaluate whether the quality-of-care indicators had improved since the introduction of the TB medical information system.

This study had two objectives:

1. To assess the completeness and concordance of data in both the paper-based and electronic records in individuals with drug-sensitive pulmonary TB in two regions of the Kyrgyz Republic who were registered between June 2021 and May 2022.
2. To compare the time from diagnosis to initiation of TB treatment and programmatic TB treatment outcomes in persons with drug-sensitive pulmonary TB in two regions of the Kyrgyz Republic who were registered in the paper-based system between January and December 2019 and in the electronic register system between June 2021 and May 2022, respectively.

## 2. Materials and Methods

### 2.1. Study Design

This was a retrospective cohort study that utilized secondary data collected routinely by the national TB control program.

### 2.2. Setting: General and Specific

This study was conducted in the Kyrgyz Republic, a landlocked country in northeastern Central Asia that was formerly part of the Soviet Union. The population is 7 million, with 1.1 million living in the capital city of Bishkek [11]. The country is divided into seven administrative regions [12], and its economy is mainly based on agriculture and mining.

The public health system in the Kyrgyz Republic is divided into three levels: central (tertiary) hospitals, regional hospitals, and polyclinics. Provincial and central hospitals are primarily referral centers for complex medical cases.

TB is a significant problem in the country. The number of newly diagnosed people with tuberculosis in the country had been falling for some years up to 2019, when 5096 cases (78.9 per 100,000 population) were diagnosed. There was a sharp decline in 2020 during the COVID-19 pandemic, with only 3518 new cases registered. This increased slightly to 3877 in 2021.

Private for-profit facilities are authorized to provide initial TB diagnosis, but all people identified as having the disease are required to be referred to public facilities for treatment under the national TB program. Inpatient and outpatient TB clinics are also available in each of the seven regions for initial diagnosis.

#### Specific Setting

People with symptoms of TB were diagnosed using sputum microscopy, GeneXpert MTB/RIF assay, and X-ray. If a sputum test was positive, the result was recorded. Prior to 2020, the information was only entered in the paper-based TB01 record, but since the introduction of the electronic register, the result and individual details are now entered in both the paper-based record and electronic TB register. Registration of people with TB was usually carried out in the outpatient department registry by a nurse using an electronic medical information system and a paper-based record. After the data were saved, an electronic medical record was automatically generated. This medical record, referred to as the “patient card”, contains all the relevant information about the people with TB and their diagnoses. The nurse sent the individual’s electronic record to the attending physician. All further TB follow-up was documented in both the paper-based and electronic registers.

### 2.3. Study Population

**Figure 1:**
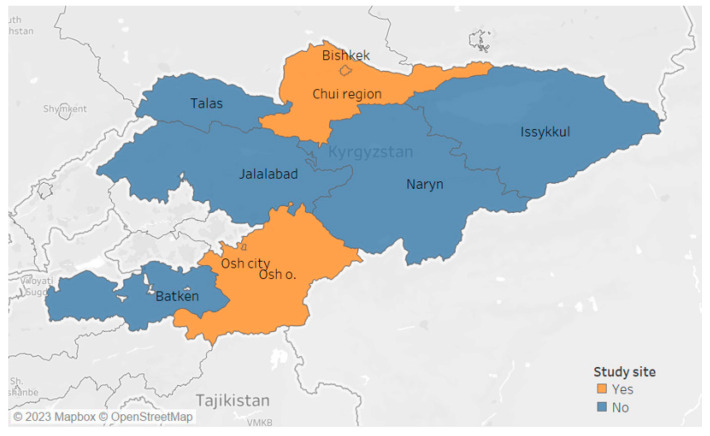
Regions of Kyrgyz Republic.

The study population included individuals with drug-sensitive pulmonary tuberculosis in the Chuy and Osh regions of the Kyrgyz Republic and Osh and Bishkek cities who were registered in the paper-based system between January and December 2019 and in the electronic system and the paper-based system between June 2021 and May 2022. A visual representation of the country’s geographical distribution is presented in Figure 1.

### 2.4. Definitions

- Paper-based records—the TB01 form used for all outpatients with TB.
- Electronic TB register—National database using the TB01 form for all persons diagnosed with TB since June 2021.
- Completeness of the records was measured as the proportion of data fields that were completed out of the total number of data fields for each data variable, and the percentage of data fields completed was also calculated for each variable.
- Concordance of records was assessed by comparing the number and percentage of fields with the same value, such as “male”, in the two data sources (the paper-based and the electronic records) for specified variables.

### 2.5. Study Sites

Data from two regions and two cities were obtained. The storage site of paper records depended on where the individual had completed treatment. This could be either at the primary level (family medicine center—FMC) or the hospital level. These regions and cities were chosen based on their feasibility and convenience, as they had high-density populations that facilitated data collection.

### 2.6. Data Variables Collected for Each Objective

#### 2.6.1. Data Variables Shown in Relation to Study Objectives

Objective 1: To assess the completeness and concordance of the data on people with drug-sensitive pulmonary tuberculosis in the Kyrgyz Republic registered between June 2021 and May 2022 in the electronic and paper-based records. Variables included individual demographics, clinical characteristics, and treatment outcomes.

Objective 2: To compare the time from diagnosis to treatment initiation and programmatic TB treatment outcomes among people with drug-sensitive pulmonary TB registered from January to December 2019 (paper-based registration) and from June 2021 to May 2022 (electronic registration). Variables included dates of diagnosis, dates of treatment initiation, and treatment outcome.

#### 2.6.2. Data Collection and Validation

The main investigator extracted data for this study from both a sample of the paper records in the health organization archives and the national electronic TB register. Convenience sampling was performed, and 25% (every fourth recorded person until the total number of cases reached 350 records) of the paper-based records from the years 2019–2022 were selected and included in the study. Paper records were obtained from FMCs and hospitals for the specified time period. All data from the national electronic TB register within the same time frame were exported and used in the study. Table 1 shows how the different data sets were compared to address the two objectives.

### 2.7. Analysis and Statistics

#### 2.7.1. Data Analysis

The data from the paper-based TB-01 journal for the 2019 period and the paper-based register during the electronic registration period (2021–2022) were entered into a separate structured Excel file using a single-entry method that included checking all entries to minimize data entry errors. Data were manually transferred into the EpiData database from the paper forms. Outlier and missing data were cross-checked with paper forms to ensure quality of data. Afterward, clean data were merged with the extracts from the national electronic TB register. The data were analyzed using STATA^®^ software (version 16.0, copyright 1985–2019 StataCorp LLC, College Station, TX, USA). Completeness was analyzed as the frequency and proportion of completed variables among all individuals registered in the electronic record. Concordance for each variable was analyzed by assessing the frequency and proportion of people with TB with the same information appearing in both the paper-based and electronic registers.

Baseline demographic and clinical characteristics of people with TB were summarized using frequencies and percentages, stratified by the pre- and post-electronic system periods. The baseline demographic and clinical characteristics across the two time periods were compared using a chi-square test.

To compare the time taken from symptom onset to diagnosis and diagnosis to initiation of TB treatment across the two time periods, this study used either Student’s *t*-test or the Mann–Whitney test depending on the distribution of continuous data. TB treatment outcomes were categorized as either successful or unsuccessful. Individuals with recorded outcomes of cure or treatment completion were classified as having a “successful outcome”, while persons who died, were lost to follow-up, experienced treatment failure, or were not evaluated were classified as having an “unsuccessful outcome”. The study used the chi-square test to compare the proportions with successful outcomes across the two time periods, as well as to compare the proportion of “not evaluated” cases in the two time periods.

#### 2.7.2. Sample Size Calculation

For objective 1: The sample size to assess completeness and concordance of the paper-based records with the electronic system for the same records was determined using a 95% confidence interval with 80% power and assuming a relative 50% reduction in the proportion of people with TB with “not evaluated” in electronic register (7%) compared with paper-based register (14%). At the ratio of 1:1 of unexposed to exposed, data needed to be extracted from 325 people with TB from both electronic and paper-based registers.

For objective 2: A sample size of 350 in each group was calculated to compare the change in the proportion of treatment outcomes between 2019 (paper-based registration) and 2021 (electronic registration period). The sample size was determined using a 95% confidence interval with 80% power and assuming a relative 40% reduction in unsuccessful outcomes in electronic period (12%) compared with paper-based period (20%). Systematic random sampling with a sampling interval of four was used to select participants from the 2019 period, and the required sample size was achieved.

## 3. Results

This study analyzed data from 302 individuals to assess the completeness and concordance of the demographic and clinical details of people with TB between the paper-based and electronic TB registers in 2021–2022. Mandatory fields were complete in both registers, while nonmandatory fields had varying levels of completeness. The lowest completion rate was observed for the number of medication side effects reported (26.8%). The date fields (i.e., date of symptom onset, diagnosis, TB treatment initiation, and the first visit to a TB clinic) had the highest completeness rates (85.1–93.0%) and concordance rates (83.2–91.1%) between the two registers. Concordance was high for most variables, including sex, age, date of birth, case definition, treatment outcome, height, weight, and location of the disease. The 302 individuals had their sex recorded electronically, but of these, only 257 had it recorded in the paper record, making it 85% complete. In 254 out of the 257 records, there was agreement in the recorded sex between the electronic and paper records. This indicates a concordance (agreement) rate of 98.85%. However, there was lower concordance observed between the two registers for reporting the number of medication side effects (see Table 2). We found that 23/350 (7%) paper records were not identified in the electronic register.

In addition, this study assessed the timing of steps between symptom onset and initiation of treatment, and also the outcomes of treatment between the two groups, the pre-implementation period (2019) or paper-based system and the post-implementation period of 2021–2022 using the electronic system. We found that the time taken from symptom onset to diagnosis was similar in both groups, with a median of 25 days in the paper-based system and 27 days in the electronic register system. The median time taken from symptom onset to treatment initiation was similar between the two groups, with a median of 29 days in the paper-based system and 30 days in the electronic register system. However, the percentage of individuals who initiated treatment on the day of diagnosis was significantly higher in the electronic register system (80.3%) compared with the paper-based system (57.1%) (*p* < 0.001) (see Table 3).

This study also compared the treatment outcomes of people with TB during the paper-based and electronic registration periods. The results showed that the proportion of successful outcomes (including cured and treatment-completed persons) was similar in the paper-based and electronic records groups (81.4% vs. 79.7%, *p* = 0.284). There were some significant differences in the subcategories of unsuccessful outcomes. Specifically, the electronic group had a higher proportion of people with TB who were lost to follow-up (5.7% vs. 2.0%, *p* < 0.001), died (5.1% vs. 1.7%, *p* < 0.001), or failed treatment (4.6% vs. 0.6%, *p* < 0.001) and had a significantly lower proportion of people with outcomes that were not evaluated or recorded (4.8% vs. 14.3%, *p* < 0.001) (Table 4).

## 4. Discussion

This study’s findings provide equivocal support for the implementation of electronic register systems in TB management. It aimed to assess the completeness and concordance of the demographic and clinical details of people with TB between paper-based and electronic TB registers. Our results showed high completeness and concordance for most variables, with some variations in nonmandatory fields. These findings are consistent with previous research on drug-resistant tuberculosis conducted in South Africa [13]. These authors found that electronic systems had higher levels of data completeness and concordance, suggesting an advantage of implementing electronic TB registers for reliable and accurate information. However, we acknowledge that determining the exact accuracy between paper and electronic records is complex. In the context of our study, both paper and electronic records underwent meticulous data entry processes, with more attention directed towards the electronic database due to familiarity and ease of use among healthcare providers. The finding that 7% of our sample of paper records was not found on the electronic database raises issues about the completeness of case recording and how quality is monitored. Despite this finding, it is expected that these individuals received their TB treatment.

This study’s results provide some insights into the completeness and consistency of demographic and clinical data between the paper-based and electronic TB registers. These findings are applicable to health facilities providing services to individuals with drug-sensitive pulmonary TB, as the study offers a preliminary assessment of the data quality of both record-keeping systems. Others [10] have found that when running both a paper-based and an electronic system, the paper system was detrimental. They recommended that one system should be chosen.

Another study that investigated electronic TB registers [14] demonstrated the potential of electronic systems in achieving high levels of data sensitivity, completeness, and agreement. These findings highlight the potential value of implementing electronic systems to replace paper-based systems, as they provide reliable and accurate information, ultimately improving TB management so long as there is adequate attention to data quality.

Our study found that medication side effects were reported in only a small proportion of cases in both the electronic TB register and paper-based records. We were unclear about what a blank field meant. This highlights the limitation of leaving the variable field blank instead of recording “0” when there are no side effects, which can result in incomplete and inaccurate data.

While there was no significant difference in the median time taken from symptom onset to diagnosis or treatment initiation between the paper-based and electronic registers, the electronic register system had a significantly higher proportion of people with TB who initiated treatment on the day of diagnosis. This observation is in line with a previous study that showed a 22% increase in the initiation of HIV patients enrolled for ART after transitioning from a paper-based system [9]. Initiating treatment on the day of diagnosis is a crucial component of early and effective TB management, highlighting the potential benefit of electronic TB registers in improving treatment outcomes. As we compared two different time periods, it may be that there are other factors that had changed that reduced the time to initiation of treatment.

The electronic register had a higher proportion of people with TB who were recorded as lost to follow-up, died, or failed treatment compared with the paper-based system, but a lower proportion where the outcome was not evaluated or recorded. One possible explanation for this finding could be that the electronic register system allowed for more accurate and comprehensive documentation of treatment. It may well be that those whose outcomes were “not evaluated” had unsuccessful outcomes such as death. This finding supports the belief that the electronic register has improved the accuracy of TB treatment outcome documentation. These findings highlight the programmatic realities of managing drug-sensitive TB and the importance of accurate and complete documentation in tracking treatment outcomes.

The strengths of this study include the use of routine data from the national TB program. The data were collected by a person with considerable experience in the program and with the data. The number of records reviewed for the second objective, 302/350 (86%), met the requirement for the sample size. Finally, the conduct and reporting of the study were in accordance with the STROBE (strengthening the reporting of observational studies in epidemiology) guidelines.

Limitations of the study include the following. The data were manually entered into the study database from paper forms. While efforts were made to ensure the quality of the data by cross-checking for outliers and missing data with the paper forms, there is still a possibility of human error during the data entry process. We had no control over the initial data entry and collection process of the national electronic TB register, which introduces a potential limitation in terms of data accuracy and completeness. Only 302/325 (93%) of the required sample size was achieved for the second objective and this was due to records not being found on the electronic system. This raises concerns about the accuracy of the initial recording of electronic data, and further work is required to understand and correct this anomaly. Only two regions of the country were sampled. These regions are thought to have better records than other regions, and so it is not clear how generalizable the results are to other parts of the country. The characteristics of people with TB could also differ in separate cohorts, as data were collected in 2019 and 2021–2022 for the first objective that assessed time to treatment initiation and treatment outcome.

Overall, our study suggests there might be some benefits of implementing an electronic TB register system for the care of people with TB in the Kyrgyz Republic, but that effective data monitoring systems would need to be put in place to ensure the completeness of individual recordings and also that the records are fully and accurately completed. The use of this system could be a reliable and effective alternative to paper-based records, providing accurate demographic and clinical data on people with TB. While the findings suggest that the electronic TB register is a valuable tool for TB care, mechanisms to regularly evaluate data quality and provide feedback to users should be put in place. There is a need to undertake a comprehensive and national-level review of both paper-based records and the electronic recording system to guide informed decisions on the current dual TB recording systems.

## Figures and Tables

**Table 1:** How TB paper and electronic records were compared to assess changes in time to treatment initiation and outcomes, and also to assess completeness of records in the TB program in Kyrgyzstan.

	Records Compared for Time from Diagnosis to Treatment Initiation and Outcomes	
	Paper records: January to December 2019	
Records compared for completeness and concordance	Electronic records: June 2021 to May 2022	Paper records: June 2021 to May 2022

**Table 2:** Completeness and concordance of demographic and clinical details of people with TB between the paper-based TB register and electronic TB register in Kyrgyzstan during 2022.

Variable	Completeness ^+^				Concordance *		
	Electronic		Paper-Based				
	*n*	(%)	*n*	(%)	Total	*n*	(%)
Total people with TB	302		302				
Auto-calculated fields							
Sex	302	(100)	257	(85.1)	257	254	(98.8)
Age	302	(100)	237	(78.5)	237	225	(95.0)
Date of birth	302	(100)	281	(93.0)	281	272	(96.8)
Mandatory fields							
Date of symptom onset	302	(100)	257	(85.1)	257	234	(91.1)
Date of diagnosis	302	(100)	259	(85.8)	259	217	(83.8)
Date of TB treatment initiation	302	(100)	281	(93.0)	281	242	(86.1)
Date of the first visit to a TB clinic	302	(100)	270	(89.4)	270	239	(88.5)
Case definition	292	(96.7)	261	(86.4)	261	260	(99.9)
Date of treatment outcome	302	(100)	209	(69.2)	209	174	(83.2)
Treatment outcome	302	(100)	229	(75.8)	229	224	(97.8)
Nonmandatory fields							
Height	284	(94.0)	263	(87.0)	259	253	(97.6)
Weight	289	(95.7)	261	(86.4)	259	255	(98.4)
Location of the disease	290	(96.0)	247	(81.8)	247	247	(100)
Number of medication side effects reported	81	(26.8)	41	(13.6)	41	35	(85.4)

**^+^** Completeness—percentage of records with the stated field completed. * Concordance—the same value entered in both the paper and electronic records.

**Table 3:** Comparison of time from symptoms to initiation of TB treatment among people with drug-sensitive TB initiated on anti-TB treatment in Bishkek pre- (2019) and post- (2021) implementation of an electronic register system in Kyrgyz Republic.

Characteristics	Paper-Based		Electronic		*p* Value ^$^
	Median	(IQR)	Median	(IQR)	
Total	350		350		
Symptom onset to diagnosis in days	25	(5–49)	27	(13–45)	0.210
Diagnosis to treatment initiation in days	0	(0–1)	0	(0–0)	<0.001
Symptom onset to treatment initiation in days	29	(13–48)	30	(16–32)	0.056
Number (percentage) initiated on treatment *					
On the day of diagnosis	200	(57.1)	281	(80.3)	<0.001
Second day of diagnosis	33	(9.4)	14	(4.0)	0.004
Third day of diagnosis	17	(4.9)	8	(2.3)	0.070
Fourth day or later of diagnosis	100	(28.6)	47	(13.4)	<0.001

* Percentages calculated with the total number of people with TB as the denominator. ^$^ Chi-square test was used to compare the proportions and Mann–Whitney U test was used for comparing the median time taken across two groups. Abbreviations: TB—tuberculosis; IQR—interquartile range.

**Table 4:** Comparison of TB treatment outcomes among people with drug-sensitive TB initiated on anti-TB treatment in Bishkek pre- (2019) and post- (2021) implementation of an electronic register system in Kyrgyz Republic.

Treatment Outcomes *	Paper-Based		Electronic		*p* Value ^$^
	*n*	(%) *	*n*	(%) *	
Total	350		350		
Successful Outcomes	285	(81.4)	279	(79.7)	0.284
Cured	105	(30.0)	88	(25.1)	0.076
Treatment completed	180	(51.4)	191	(54.6)	0.203
Unsuccessful Outcomes					
Lost to follow-up	7	(2.0)	20	(5.7)	<0.001
Death ^#^	6	(1.7)	18	(5.1)	<0.001
Failure	2	(0.6)	16	(4.6)	<0.001
Not evaluated/recorded	50	(14.3)	17	(4.8)	<0.001

* Percentages calculated with the total number of people with TB as the denominator. ^#^ Death includes death due to TB and other causes. ^$^ Chi-square test was used to compare the proportions. Abbreviations: TB—tuberculosis; IQR—interquartile range.

## Data Availability

Requests to access these data should be sent to the corresponding author.

## Open Access Statement and Disclaimer

In accordance with WHO’s open-access publication policy for all work funded by WHO or authored/co-authored by WHO staff members, WHO retains the copyright of this publication through a Creative Commons Attribution IGO license (accessed on 13 August 2023) which permits unrestricted use, distribution, and reproduction in any medium provided the original work is properly cited. There should be no suggestion that WHO endorses any specific organization, products or services. The views expressed in this article are those of the authors and do not necessarily reflect those of their affiliated institutions. The use of the WHO logo is not permitted. This notice should be preserved along with the article’s original URL.
